# The imperative of cholecystitis: understanding and prioritizing gallbladder perforation

**DOI:** 10.1097/JS9.0000000000001462

**Published:** 2024-04-10

**Authors:** Jintang He, Zheng Li, Jie Huang

**Affiliations:** a Department of Cardio-Thoracic Surgery, The Yunnan Clinical Medical Center for Infectious Diseases, The Third People’s Hospital of Kunming; b Department of Hepatopancreatobiliary Surgery, The Second Affiliated Hospital of Kunming Medical University, Kunming, People’s Republic of China

*Dear Editor*,

I read with great interest the article by Lee *et al*.^1^, recently published in the International Journal of Surgery, which presents a retrospective cohort study on the impact of gallstones on the development of gallbladder perforation (GBP) in patients with acute acalculous cholecystitis (AC) and calculous cholecystitis (CC). The study’s 10-year single-center experience analyzed 4497 patients who underwent cholecystectomy, revealing a significantly higher incidence of GBP in the AC group (5.6%) compared to the CC group (1.0%). The authors identified AC as a significant risk factor for GBP, with patients experiencing a fivefold higher risk than those with CC. Other factors associated with an increased risk of GBP included older age, male sex, comorbidities, poor performance status, and concurrent acute cholangitis. The study found that early cholecystectomy significantly reduced GBP-related morbidity and mortality. The findings underscore the importance of prompt clinical attention and surgery to mitigate the complications associated with GBP. However, I would like to raise a few points for discussion.

Firstly, in the ‘Results’ section under ‘Study Population and Baseline Characteristics,’ the authors state that the duration from hospital admission to surgical intervention was significantly longer in the AC group compared to the CC group (3.7±5.8 vs. 2.7±3.0 years, *P*<0.001). However, Table 1 provides data on ‘Duration from Admission to Cholecystectomy (days, mean±SD)’ as (3.7±5.8 vs. 2.7±3.0, *P*<0.001) and ‘Duration from Symptom to Admission (days, mean±SD)’ as (3.3±3.9 vs. 4.0±4.5, *P*<0.001). Could the authors clarify the source of the data on the duration from hospital admission to surgical intervention (3.7±5.8 vs. 2.7±3.0 years, *P*<0.001) as stated? Or could this be a typographical error? Similarly, in Table 2, the ICU stay for the AC group was 5.3±7.9 days, while for the CC group, it was 3.6±3.4 days. However, the total hospital stay for the groups was 9.0±7.4 days for the AC group and 8.1±52.5 days for the CC group. The large SD in the total hospital stay for the CC group is intriguing and raises the question of whether certain patients experienced severe postoperative complications contributing to this variance. If so, would the authors consider providing a separate clarification for such cases?

Secondly, bile duct injuries during cholecystectomy are indeed a surgeon’s nightmare^2^. In the authors’ study, within the CC group, there were two cases of biliary stricture. As indicated in Table 4, both instances occurred in the delayed cholecystectomy group. Could the authors elucidate whether these strictures are related to surgical misadventures or are a consequence of the Mirizzi syndrome?

Thirdly, I find it intriguing that in the authors’ research, there was only one case of Type III perforation. However, the manuscript does not specify whether this perforation resulted in a cholecystocolonic fistula or a cholecystoduodenal fistula, as the management of such cases is particularly challenging, and any misstep could lead to severe complications. In my review, I have observed that the authors seem to have overlooked a specific variant of acute cholecystitis—xanthogranulomatous cholecystitis. This is significant because patients with this condition may exhibit imaging suggestive of GBP prior to surgery^3^. Therefore, it would be beneficial for the authors to clarify whether cases of xanthogranulomatous cholecystitis were included in their study. This information is crucial for ensuring the comprehensiveness and accuracy of the research findings and for preventing potential biases in patient selection that could compromise the validity of the conclusions.

In conclusion, the article by Lee *et al*. presents a well-conducted study with accurate information and logically sound arguments. The findings highlight the importance of considering AC as a risk factor for GBP and suggest that early surgical intervention may be beneficial in reducing related complications. The study contributes valuable insights to the field of surgical gastroenterology and warrants consideration in the management of patients with acute cholecystitis.

## Ethical approval

This paper is a correspondence and does not require ethical approval.

## Consent

This paper is a correspondence and does not require consent.

## Sources of funding

This work was supported by the Academic Leadership Training Program at the Second Affiliated Hospital of Kunming Medical University (Grant No. RCTDXS-202309) and the Kunming Medical University’s teaching research project (Grant No. 2023-JY-Y-059).

## Author contribution

J.H.: conceptualisation, data curation, formal analysis, investigation, and methodology; Z.L.: writing–original draft preparation; J.H.: data curation, supervision, and writing–reviewing and editing. All authors approved the final version of the manuscript.

## Conflicts of interest disclosure

Not applicable.

## Research registration unique identifying number (UIN)

Not applicable.

## Guarantor

Jie Huang.

## Data availability statement

Data availability is not applicable to this article as no new data were created or analyzed in this study.

## Provenance and peer review

Not applicable.

## References

[1] Lee KJ Park SW Park DH . Gallbladder perforation in acute acalculous vs. calculous cholecystitis: a retrospective comparative cohort study with 10-year single-center experience. Int J Surg 2024;110:1383–1391. 38079596 10.1097/JS9.0000000000000994 PMC10942242

[2] Li J Xu D Huang J . Biliary tract injury after cholecystectomy: a surgeon’s worst nightmare. Int J Surg 2023;109:2535–2536. 37216229 10.1097/JS9.0000000000000474 PMC10442093

[3] Frountzas M Schizas D Liatsou E . Presentation and surgical management of xanthogranulomatous cholecystitis. Hepatobiliary Pancreat Dis Int 2021;20:117–127. 33536138 10.1016/j.hbpd.2021.01.002

